# Concussion-Related Cognitive and Lipid Changes in Retired UK Rugby Players Study

**DOI:** 10.3390/ijms262211002

**Published:** 2025-11-13

**Authors:** Norah Alanazi, Toni Robinson, Ian Entwistle, Karen Hind, Paul Chazot

**Affiliations:** 1 Department of Biosciences, Wolfson Research Institute for Health and Wellbeing, Durham University, Durham DH1 3LE, UK; norah.alanazi@durham.ac.uk (N.A.); toni.g.robinson@durham.ac.uk (T.R.); 2 Wolfson Research Institute for Health and Wellbeing, Durham University, Durham TS17 6BH, UK; karenhindphd@gmail.com

**Keywords:** lipids, biomarkers, concussion, brain injury, sport, neurodegenerative diseases

## Abstract

Long-term effects of concussions, particularly in contact sport athletes, have been linked to changes in neuronal health. Lipid dysregulation has emerged as a potential contributor to neuronal injury and may serve as a measurable biomarker of brain pathology. This study investigated cognitive scores and serum lipid biomarkers in retired rugby players with a history of concussion to assess their association with concussion exposure. Serum levels of 24-hydroxycholesterol (24-HC), 25-HC, 27-HC, total triglycerides, and ceramide were compared between retired rugby players with a history of repeated concussions (n = 26) and non-contact sport controls (n = 19). ELISA-based quantification and statistical analyses identified significant group differences. Concussed athletes exhibited significantly lower serum 24-HC and significantly higher levels of 27-HC, triglycerides, and ceramide compared to controls, while no significant difference was observed for 25-HC. These findings indicate that repeated concussion is associated with reductions in cognitive performance and persistent alterations in serum lipid profiles. The observed lipid changes, particularly in 24-HC, 27-HC, ceramide, and triglycerides, may serve as measurable biomarkers of concussion-related biochemical alterations, providing a foundation for future studies aimed at monitoring neurological health in at-risk populations.

## 1. Introduction

Rugby, a well-known contact sport, carries a notably high risk of physical injury—particularly concussions—due to its inherently aggressive nature, which includes frequent and repeated tackles and collisions [1]. Concussions are a form of traumatic brain injury resulting from rapid head movements involving acceleration and deceleration. They often manifest as brief loss of consciousness, impaired reflexes, or amnesia [2]. Typical symptoms include headaches, dizziness, sleep irregularities, balance issues, disorientation, cognitive disturbances, and mood changes [3]. These injuries are particularly concerning because of their association with long-term deficits in cognition, motor abilities, and mental health [4,5,6].

Previous research indicates that recurrent concussions may exert cumulative effects, heightening the risk of subsequent brain injuries and contributing to the progressive decline in neurocognitive functions. Tsushima et al. [7] found that athletes with a history of concussion were three to five times more likely to experience additional concussive events compared to those with no prior history. Moreover, experiencing concussions can significantly impair cognitive functions such as memory, executive function, and motor coordination. Retired athletes across various sports who have experienced concussions tend to report greater cognitive difficulties [8]. In a study by Kerr et al. [9] involving 204 former collegiate football players (84.3% with a history of concussion), the researchers explored the relationship between previous concussions and negative health outcomes. The study identified significant associations between concussion history and multiple negative health outcomes, encompassing diminished physical and mental well-being, higher prevalence of depressive symptoms, and a greater likelihood of alcohol dependence.

While physical activity generally promotes overall health, there are instances where the potential risks may surpass the advantages of engaging in exercise. In this regard, there is growing concern about the health and well-being of former contact sport athletes, particularly in relation to the potential long-term effects of concussions and other brain injuries. For example, former professional and collegiate athletes in sports such as soccer and American football have been found to experience significantly higher rates of amyotrophic lateral sclerosis (ALS) [10]. Lehman et al. [11], in a comprehensive mortality analysis of 3439 National Football League (NFL) players, reported that the incidence of deaths from neurodegenerative diseases—such as Alzheimer’s disease and amyotrophic lateral sclerosis (ALS)—was approximately threefold higher than that of the general population. In an earlier investigation, Guskiewicz et al. [12] surveyed 2552 retired professional football players using a general health questionnaire, revealing that 61% had sustained at least one concussion and 24% had experienced three or more during their playing careers. The study found that players with a greater history of repeated concussions were at increased risk for developing dementia-related conditions. Additional research has consistently shown a strong association between traumatic brain injury (TBI) and elevated risk of cognitive decline and neurodegenerative disorders, including Alzheimer’s disease. Regrettably, most neurodegenerative diseases that lead to dementia remain undiagnosable and untreatable during their early, prodromal stages due to the lack of clinically validated biomarkers [13]. In addition, there is currently a gap in research, as no studies have specifically explored potential concussion-related biomarkers that may be associated with neurodegenerative conditions in retired athletes with a history of sports-related concussions (see the exceptional Alanazi et al., 2024) [14].

The present study investigates lipid biomarkers, conventionally linked to cardiovascular pathology, as prospective indicators of neurodegeneration in a well-characterized cohort of retired rugby players with a history of recurrent concussions, in comparison with healthy controls. The lipid biomarkers examined include 24S-hydroxycholesterol (24-HC), 25-hydroxycholesterol (25-HC), 27-hydroxycholesterol (27-HC), ceramide, and total triglycerides.

Lipids are a diverse group of organic molecules that play essential roles in cellular structure, energy storage, and signaling pathways (Fahy et al., 2009) [15]. Remarkably, the brain contains one of the highest concentrations of lipids in the human body—second only to adipose tissue—with lipids accounting for over 50% of its dry weight (Bruce et al., 2017) [16]. These molecules are fundamental to key neurological functions such as axonal insulation, neurite outgrowth, and synapse formation. Consequently, disruptions in lipid metabolism have been increasingly linked to neurodegenerative processes and brain disorders (Cermenati et al., 2015) [17]. Additionally, brain lipid regulation is closely intertwined with oxidative stress, neuroinflammation, and the maintenance of energy balance. Dysregulated lipid metabolism in neuroglial cells can adversely affect normal neuronal function and signaling (Yang et al., 2022) [18]. Following traumatic brain injury (TBI), measurable alterations in lipid profiles have been detected in peripheral fluids such as blood, plasma, and serum, highlighting their potential as accessible biomarkers (Nessel et al., 2021) [19]. In this context, a deeper understanding of lipid dynamics may not only enhance the diagnosis of concussion-related injuries but also inform early intervention strategies for mitigating long-term neurodegenerative risks.

Oxysterols—oxidized derivatives of cholesterol such as 24-HC, 25-HC, and 27-HC—play pivotal roles in both physiological regulation and pathological progression, particularly in the context of neurodegenerative disorders, including Alzheimer’s disease (AD), Parkinson’s disease (PD), and amyotrophic lateral sclerosis (ALS) (de Freitas et al., 2021) [20]. These metabolites arise via distinct enzymatic pathways: 24-HC is predominantly synthesized in the brain through the action of cholesterol 24-hydroxylase (CYP46A1) (Sun et al., 2016) [21], 25-HC is produced in macrophages and glial cells via cholesterol 25-hydroxylase (CH25H) in response to Toll-like receptor 4 (TLR-4) activation (Romero et al., 2024) [22], and 27-HC is generated by CYP27A1, which is widely expressed across various human tissues (Marwarha and Ghribi, 2015) [23]. These distinct hydroxylation sites on the cholesterol molecule contribute to their varying cytotoxic effects on neural cells and are believed to play critical roles in the pathogenesis of neurodegeneration (Nakazawa et al., 2017) [24]. Elevated levels of 24-HC in serum have been associated with TBIs and linked to neurodegenerative conditions such as multiple sclerosis and PD. Conversely, reduced 24-HC concentrations may reflect neuronal loss and brain atrophy, as observed in AD and multiple sclerosis (Leoni et al., 2002; [25] Björkhem et al., 2013) [26]. Due to its enhanced ability to cross biological membranes, including the blood–brain barrier, 24-HC holds promise as a serum biomarker for brain injury (Lu et al., 2021) [27]. Likewise, 25-HC has been shown to induce apoptosis in cultured motor neurons, with high levels negatively impacting neuronal viability (Odnoshivkina et al., 2022) [28]. Clinical findings by Kim et al. (2017) [29] revealed significantly elevated 25-HC levels in both serum and cerebrospinal fluid (CSF) of ALS patients compared to controls, implicating its role in disease pathology. Regarding 27-HC, elevated levels have been observed in patients with mild cognitive impairment, suggesting a link to early AD development (Zhang et al., 2019) [30]. However, contrasting evidence from Wuolikainen et al. (2014) [31] showed decreased 27-HC plasma levels in ALS patients relative to controls, indicating complex and disease-specific patterns in oxysterol metabolism.

Ceramide, a bioactive lipid composed of a sphingosine backbone linked to a fatty acid, is a central component of sphingolipid metabolism and plays a critical role in cellular signaling and homeostasis (Mencarelli and Martinez–Martinez, 2013) [32]. The regulation of intracellular ceramide levels is vital, as alterations in ceramide and sphingolipid profiles have been implicated in the pathophysiology of various neurological, neuroinflammatory, and age-associated disorders (Mencarelli & Martinez–Martinez, 2013) [32]. Elevated ceramide concentrations have been linked to mitochondrial dysfunction, impaired autophagic processes, and disrupted amyloid-β clearance, all of which are recognized contributors to the development and progression of Alzheimer’s disease (AD) (Chowdhury et al., 2022 [33]; Custodia et al., 2022 [34]; Tringali and Giussani, 2022) [35]. Notably, Han et al. (2002) [36] observed increased ceramide levels during the early stages of AD, with a subsequent decline as the disease advanced, suggesting a dynamic role in disease progression. Post-mortem analysis by He et al. (2010) [37] further confirmed elevated ceramide concentrations in brain tissues of AD patients compared to controls. Similarly, Fernández-Irigoyen et al. (2021) [38] reported significantly higher ceramide levels in the plasma of patients with Parkinson’s disease (PD) relative to healthy individuals. In preclinical research, Barbacci et al. (2017) [39] utilized mass spectrometry imaging to examine ceramide distribution in a rat model of controlled cortical impact (CCI), revealing elevated ceramide in damaged brain regions. In parallel, Ojo et al. (2019) [40] analyzed phospholipid alterations, including ceramide dysregulation, in mouse models of repetitive mild TBI and AD. Their findings identified both shared and unique lipidomic signatures, highlighting ceramide’s potential role in the transition from TBI to neurodegenerative disease. More recently, Liu et al. (2024) [41] demonstrated that mice with increased serum ceramide exhibited poorer cognitive performance, as evidenced by reduced discrimination indices, suggesting that circulating ceramide may serve as a viable biomarker for cognitive impairment.

Triglycerides are a class of lipids primarily involved in the storage and transport of energy. They originate from two main sources: dietary absorption through the gastrointestinal tract and endogenous synthesis by the liver. Structurally, triglycerides are composed of three fatty acids bound to a glycerol molecule, and their diversity is vast, with an estimated 6000 distinct forms based on variations in fatty acid composition and configuration (Bernath et al., 2020) [42]. Triglycerides are fundamental constituents of triglyceride-rich lipoproteins, such as chylomicrons and very-low-density lipoproteins (VLDLs), and also exist in remnant particles that persist following lipoprotein catabolism (Do et al., 2013) [43]. The relationship between serum triglyceride levels and cognitive function has been explored in several human studies. Parthasarathy et al. (2017) [44], in a cohort of 251 older adults, reported an inverse correlation between serum triglyceride levels and executive function, although no association was found with memory performance. These findings suggest that elevated triglyceride levels may selectively impair certain cognitive domains. Similarly, Bernath et al. (2020) [42] conducted a cross-sectional analysis involving 689 participants, including both Alzheimer’s disease (AD) patients and healthy controls. Their results indicated that lower triglyceride levels were associated with better cognitive outcomes and reduced brain atrophy, implying a potential protective role. Complementing this, Zhao et al. (2019) [45] assessed serum lipid profiles in a large sample of 1762 individuals aged 40 to 85. They found that higher triglyceride levels were significantly associated with cognitive decline in middle-aged men (≤55 years), further supporting a link between dyslipidemia and cognitive impairment. Despite these associations, human studies specifically examining serum triglycerides in individuals with a history of concussion or traumatic brain injury (TBI) remain lacking. However, animal models provide preliminary insights. For instance, Kuo et al. (2020) [46] demonstrated that rats subjected to TBI and fed a high-fat diet exhibited elevated triglyceride levels, which correlated with increased injury severity. Additionally, Hahnefeld et al. (2022) [47] reported that triglyceride accumulation contributed to behavioral changes post-TBI, highlighting a potential connection between altered lipid metabolism and neurobehavioral outcomes following brain injury.

Given their association with neuronal injury and degeneration, 24-HC, 25-HC, 27-HC, ceramide, and total triglycerides represent promising biomarker candidates for the early detection of neurodegenerative risk. This is particularly relevant for retired rugby players, who are frequently exposed to repeated head trauma. Early identification of oxysterol and other lipid imbalances in this population could inform timely therapeutic strategies aimed at reducing long-term cognitive and neurological burden.

## 2. Results

A total of 45 male participants were randomly selected for inclusion in this study, comprising 26 individuals with a documented history of concussion and 19 control participants without such a history. Detailed demographic and descriptive information for all participants is presented in Table 1 below.

### 2.1. Serum Biomarker Levels in Concussed and Control Groups

**Figure 1:**
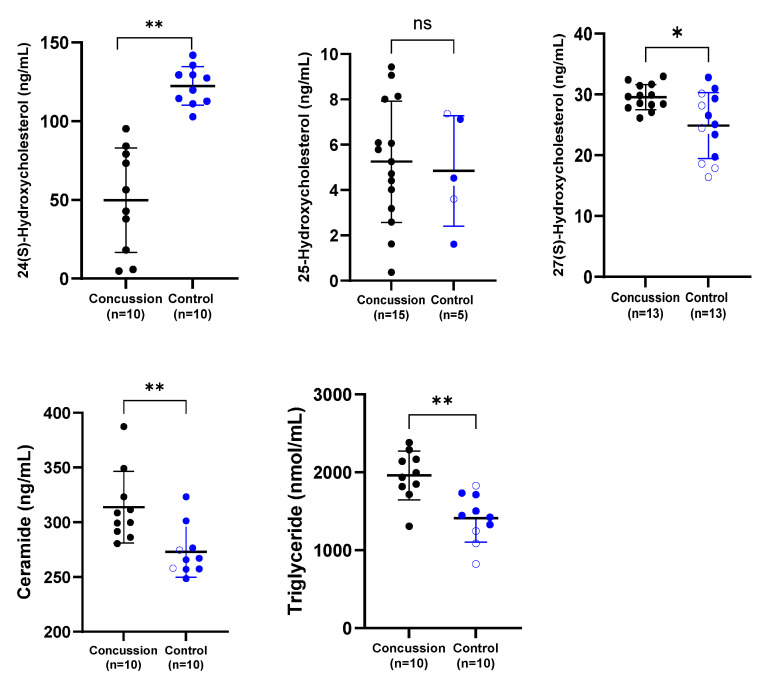
**Comparison of serum concentrations of 24-HC, 25-HC, 27-HC, ceramide, and total triglycerides between the concussion and control groups.** Each dot represents an individual participant, with bars indicating the median and range. Blue dots represent the control group, while black dots denote the concussion group. * *p* < 0.05; ** *p* < 0.01; ns = not significant.

A range of related serum biomarkers was analyzed (Figure 1). Serum concentrations of 24-HC were significantly lower in the retired rugby player group compared with the healthy control group (*p* < 0.01). In contrast, serum ceramide and total triglyceride concentrations were markedly elevated in retired rugby players with a history of multiple concussions relative to the non-contact control group (*p* < 0.01). Furthermore, levels of 27-HC differed significantly between the concussed and control groups (*p* < 0.05), whereas no significant difference was observed in serum 25-HC concentrations between the two groups.

### 2.2. Correlations Between Biomarkers

A standard Pearson’s correlation (regression) analysis was performed to examine the associations among all measured biomarkers. Unexpectedly, there were insignificant correlations between 24-HC levels and total 25-HC (R^2^ = 0.03717, *p* = 0.41); 24-HC and 27-HC (R^2^ = 0.1508, *p* = 0.09); 25-HC and 27-HC (R^2^ = 0.03090, *p* = 0.45); 25-HC and ceramide (R^2^ = 0.06039, *p* = 0.2963); 27-HC and ceramide (R^2^ = 0.06558, *p* = 0.27); 25-HC and triglycerides (R^2^ = 0.02654, *p* = 0.49); 27-HC and triglycerides (R^2^ = 0.04482, *p* = 0.37); ceramide and triglycerides (R^2^ = 0.1939, *p* = 0.052).

**Figure 2:**
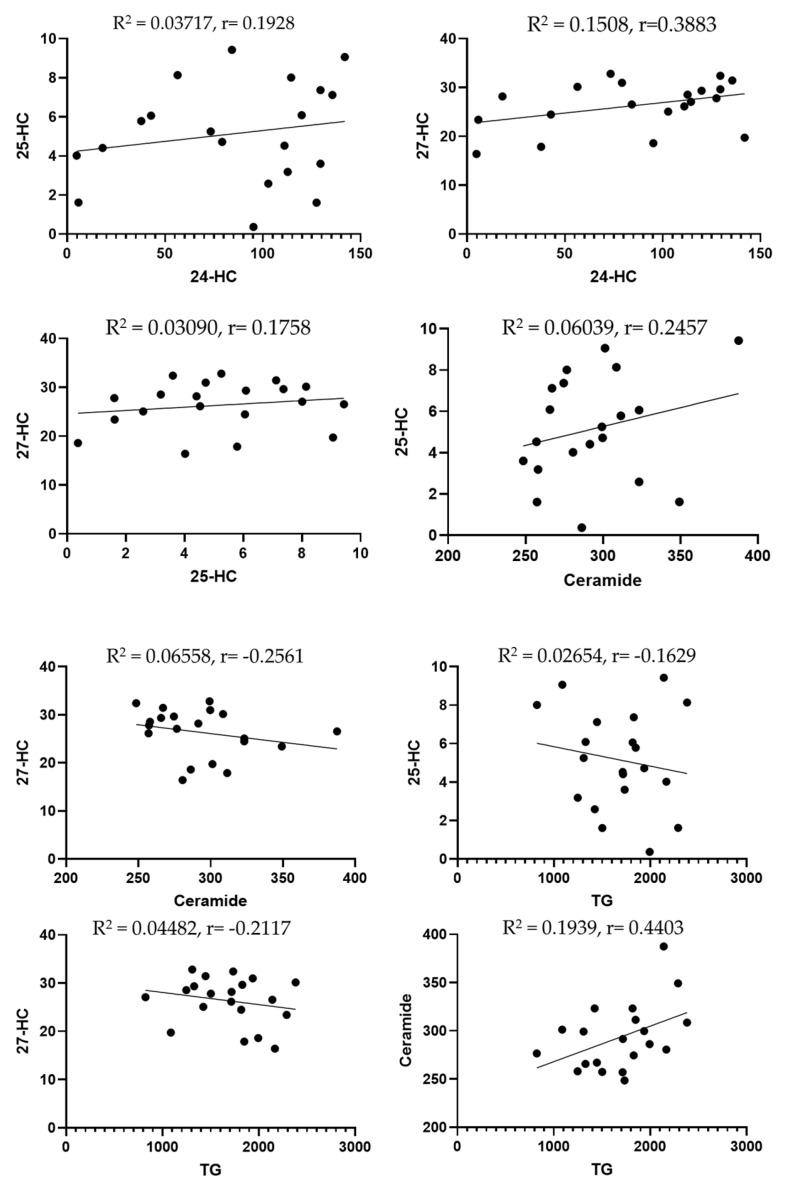

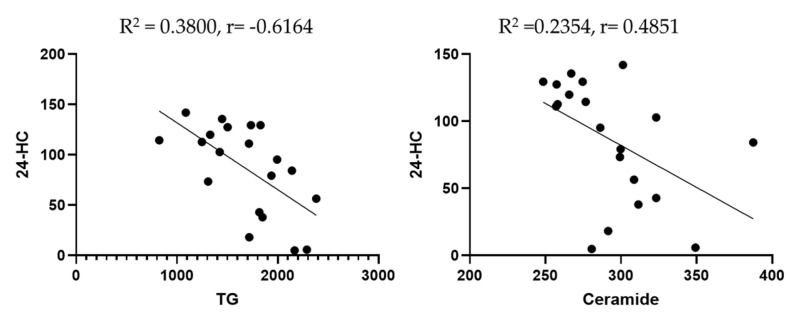
Scatter plots to highlight the correlation between lipid biomarkers. Dots are individual data points for the concentration of the substances specified on graphs of specific serum samples (n = 20 or more).

A significant inverse correlation was observed between reduced 24-HC levels and increased total triglycerides (R^2^ = 0.38, *p* = 0.003), as well as between lower 24-HC levels and elevated ceramide concentrations (R^2^ = 0.24, *p* = 0.03). (Figure 2).

### 2.3. Cognitive Parameters

The cognitive parameters tested and analyzed in this research include the raw scores for memory, verbal memory, visual memory, psychomotor speed, reaction time, complex attention, cognitive flexibility, processing speed, executive function, simple attention and motor speed. These parameters were measured using the Vital Signs Computerized Neurocognitive Assessment which was developed as a routine clinical screening tool. These tests are used by many health professionals who acknowledge the reliability and validity of the results. The tests ran as part of the assessment are sensitive to most of the causes of cognitive dysfunction or impairment. The raw test scores were provided as part of the UK Rugby Health Study.

An initial investigation of the test score results was carried out to test for significance between a group who had experienced five or more concussions, compared to a group who had suffered from none. The scores were used to calculate the neurocognitive index (NCI), which gave an overall score for neurological function. Table 2 presents the initial comparative findings between individuals with a history of five or more concussions and those with no history of concussion. Notably, the neurocognitive index, cognitive flexibility, and executive function showed significant differences between the groups. These three cognitive parameters provide a strong initial indication of concussion-related effects and offer a foundation for further analysis of broader cognitive outcomes.

## 3. Discussion

Given their established links to neurodegenerative conditions such as Alzheimer’s disease (AD) and amyotrophic lateral sclerosis (ALS), this paper assesses the concentrations of key lipid biomarkers— 24-HC, 25-HC, 27-HC, triglycerides, and ceramide—between retired rugby players with a history of concussion and matched healthy controls. These markers were examined to determine whether measurable differences exist that may serve a diagnostic purpose. This paper highlights that several lipid species may contribute meaningfully to the early detection of concussion-related pathology and the long-term risk of neurodegeneration, with potential implications for revising concussion protocols in high-impact sports such as rugby.

The findings revealed significantly reduced serum 24-HC concentrations in concussed individuals compared to the control group (Figure 1). This observation corresponds to previous studies in neurological disorders. For instance, lower 24-HC levels have been documented in older patients with multiple sclerosis (MS), Parkinson’s disease (PD), and ALS, likely reflecting neuronal loss or impaired metabolic activity in cholesterol catabolism (Lee et al., 2009 [48]; La Marca et al., 2016 [49]). Conversely, studies such as Weiner et al. (2008) [50] did not report a difference in 24-HC following TBI, although their sample involved younger individuals, which may account for this discrepancy. Overall, our findings support the hypothesis that decreased 24-HC may indicate reduced neuronal viability and suggest its potential utility as a biomarker for concussion-induced neural damage.

Regarding 25-HC, no significant group differences were observed; however, several concussed individuals exhibited elevated levels relative to controls (Figure 1). Prior research on ALS patients showed mixed outcomes—Kim et al. (2017) [29] reported increased serum and CSF 25-HC, whereas Wuolikainen et al. (2014) [31] found no significant alterations. The lack of statistical significance in our study may be attributed to the small sample size, the variability of ELISA assays, or undiagnosed head injuries in the control cohort.

Serum levels of 27-HC were found to be elevated in both the concussed and control groups (Figure 1). These findings partially mirror reports of increased 27-HC in CSF of ALS patients (Kim et al., 2017) [29], possibly due to impaired clearance or upregulated synthesis. In contrast, reduced plasma 27-HC was observed by Wuolikainen et al. (2014) [31] in ALS, suggesting divergent peripheral and central regulation mechanisms. This divergence emphasizes the need to distinguish between systemic and CNS-specific oxysterol pathways in neurodegenerative risk.

Total serum triglyceride levels were also significantly elevated in the retired rugby players compared to controls (Figure 1). This novel finding aligns with studies linking triglyceride dysregulation to brain atrophy and reduced cognitive function (Bernath et al., 2020 [42]; Zhao et al., 2019 [44]). Furthermore, elevated triglyceride concentrations have been associated with a heightened risk of dementia and AD (Tian et al., 2023) [51]. Animal studies further substantiate these associations—Kuo et al. (2020) [46] and Hahnefeld et al. (2022) [47] both reported that increased triglycerides exacerbated injury and behavioral deficits post-TBI.

Ceramide levels were notably higher in the concussed group relative to controls (Figure 1), representing another novel contribution. While ceramide has been extensively studied in AD and PD, its association with sports-related concussion has not previously been investigated. Prior work demonstrated elevated ceramide in early AD and PD cases (Han et al., 2002 [36]; He et al., 2010 [37]; Fernández-Irigoyen et al., 2021 [38]). Animal models similarly reveal ceramide accumulation in brain regions impacted by injury (Barbacci et al., 2017 [39]; Ojo et al., 2019 [40]). Most recently, Liu et al. (2024) [41] linked elevated ceramide with cognitive impairment in mice, suggesting serum ceramide as a promising marker of neural dysfunction.

With reference to the correlation between biomarkers, we found a negative correlation between 24-HC and triglycerides. Previous studies discussed the association between 24-HC and triglycerides. For instance, Dunk et al. (2024) [52] found that there is an association between 24-HC and triglycerides, suggesting that plasma oxysterols may be a modifiable risk factor for dementia. Lin et al. (2018) [53] observed that the plasma 24-HC was correlated with total plasma cholesterol (R = 0.659, *p*= 0.001), affecting the risk of age-related macular degeneration (AMD) development and progression. Though not a direct measure of triglycerides, total cholesterol correlation often implies a broader relationship across lipid fractions, including TG. Furthermore, key circulating oxysterols, including 24-HC, were found to be positively correlated with triglycerides, indicating systemic lipid interactions (Passarelli et al., 2022) [54]. These studies suggest that 24-HC levels are significantly associated with systemic lipid markers, including triglycerides. They reinforce the biological link between brain cholesterol metabolism (via 24-HC) and peripheral lipid status. We also observed a negative correlation between 24-HC and ceramide. Previous research suggested that 24-HC is substantially reduced in the presence of neurodegenerative disease. Because the final effect of a neurodegenerative process is a loss of active neural cells, a reduction in 24-hydroxylase activity, with subsequent decline in the formation of 24-HC and its lower efflux from the brain into circulation, is likely to be the outcome in these disorders (Leoni et al., 2013) [55]. Prior studies also denoted that higher levels of ceramide are associated with increased AD pathology and inflammation (Teitsdottir et al., 2021) [56]. Currently, no studies have reported direct correlations between 24-HC and ceramide levels in neurological patients or within the context of concussion/TBI. This highlights the novelty of the present study, which explores lipid biomarkers and their interrelationships as potential tools for diagnosing concussions and predicting neurodegenerative disease.

Taken together, these results suggest that lipids—particularly 24-HC, 27-HC, triglycerides, and ceramide—warrant further investigation as potential biomarkers of concussion-related neurodegeneration. Although the sample sizes for individual lipid analyses were limited due to restricted serum availability, the findings provide preliminary evidence supporting lipid dysregulation following repeated head trauma. This study represents one of the first attempts to explore lipidomics in the context of repeated concussion exposure in contact sport athletes and contributes valuable evidence for refining diagnostic strategies and long-term monitoring. Future studies can incorporate standardized neurocognitive assessments to further elucidate the relationship between lipid dysregulation and cognitive function. Also, future studies, incorporating inflammatory and glial activation markers (e.g., TNF-α, IL-1β, IL-6, GFAP, sTREM2) will be important to determine whether the observed neuronal metabolic changes occur independently of, or alongside, neuroinflammatory responses.

## 4. Materials and Methods

### 4.1. Study Design and Setting

The current research analyzed a range of selected biomarkers in retired rugby players with a history of concussion and non-contact sports controls from the UK Rugby Health Project [6]. The UK Rugby Health Project was initiated in 2016 as an extension to the inaugural New Zealand Rugby Health Project [57], and the methods and results so far have been published elsewhere [1,6,57,58]. UK Rugby Health Study. Original Ethics approval (Dr Karen Hind). Leeds Beckett University/Carnegie Research Ethics Committee 15 June 2016. Follow up for Serum markers (Prof Paul Chazot/Karen Hind) SPORT-2020-07-21T08:04:55-brbr69 Status Approved Approval date 24 July 2020.

### 4.2. Study Participants

Former male rugby players and non-contact sport athletes took part in the study and were recruited from September 2016 to December 2018 using past player/athlete associations, printed and televised media reports, word of mouth, and social media. Participants in the current study were those who had attended an in-person clinical appointment and provided a fasted blood sample. The group of concussed in this study were retired rugby players who reported 5 or more diagnosed concussions during their sporting career (n = 26), and the control group was both retired rugby players and retired non-contact sports participants with no reported concussions (n = 19).

Due to limited serum volumes and differing assay requirements, not all biomarkers could be analyzed in every participant. As a result, sample sizes varied across biomarkers (e.g., 24-HC: n = 10 per group; 25-HC: n = 15 concussed vs. n = 5 control; 27-HC: n = 13 per group; ceramide and total triglycerides n = 10 per group). This variation, together with the significant age difference observed between groups (*p* < 0.05), is acknowledged as a limitation of the present study.

The severity of the individual concussions was not monitored, and only the group with over 5 concussions was explored in this initial study to explore the extremes in the first instance. Further studies will follow to meet these limitations.

A general health questionnaire was used to gather information regarding engagement in rugby, injuries, including concussions, present health and well-being, and height and weight measurements [1,6]. The questionnaire was accessible online from September 2016 to December 2018.

### 4.3. Biomarker Assays

This project investigated concentration levels of serum (24-HC, 25-HC, 27-HC, ceramide, and total triglycerides) for the highly concussed cohort and the non-concussed UK athletes [59,60,61].

### 4.4. ELISA Assays

Assays performed using serum followed manufacturer instructions for 24-HC (abcam, Cambridge, UK, catalog no: ab204530), 25-HC (BT lab, Shanghai, China, catalog no: EA0143Hu), 27-HC (abbexa, Cambridge, UK, catalog no: abx257403), ceramide (AFG Scientific, Arlington Heights, IL, USA, catalog no: EK710698), and total triglycerides (abbexa, Cambridge, UK, catalog no: abx257659).

### 4.5. The CNSVS Battery

Cognitive parameters were measured using the Vital Signs Computerized Neurocognitive Assessment, which was developed as a routine clinical screening tool (Gualtieri & Johnson, 2006) [62]. The clinical evaluation battery is made up of seven tests: verbal memory (VBM), visual memory (VIM), finger tapping (FTT), symbol digit coding (SDC), the Stroop Test (ST), a test of shifting attention (SAT) and the Continuous Performance Test (CPT). These tests are used widely by neuropsychologists with acknowledgment of their reliability and validity. The tests are also acknowledged to be sensitive to most of the causes of cognitive dysfunction or impairment. The test scores were provided by Professor Paul Chazot as part of the UK Rugby Health Study.

### 4.6. Verbal and Visual Memory Test (VBM and VIM)

The verbal memory test is a variation of the Rey Auditory Verbal Learning Test (Rey, 1964; Taylor, 1959) [63,64]. In the Vital Signs CNS version of the test, 15 words are shown one by one, every two seconds, on a screen (Gualtieri & Johnson, 2006) [62]. The participant is asked to remember the 15 words before being shown a list of 30 words. Among the 30 words, the original 15 are mixed randomly. When the participant recognizes one of the 15 target words, they press the space bar. After the other 6 tests, the participant is shown 30 words, again with the 15 target words mixed in, and asked to recognize them.

The visual memory test is also a variation of a Rey test where it uses geometric figures rather than words (Rey, 1964; Taylor, 1959) [63,64]. A total of 15 figures are shown, and the participant is asked to remember them before being shown 30 figures (Gualtieri & Johnson, 2006) [62]. Among the 30 figures, the original 15 are mixed randomly. When the participant recognizes one of the 15 target figures, they press the space bar. After 5 other tests, the participant is shown 30 figures, again with the 15 target figures mixed in, and asked to recognize them.

The scores of verbal memory and visual memory are recorded separately, as well as being summed to generate a raw memory score.

### 4.7. The Finger Tapping Test (FTT)

The finger tapping test is a simple test that asks the participant to tap the space bar as many times as they can in 10 s (Gualtieri & Johnson, 2006) [62]. The test consists of one practice, then three runs. It is then repeated with testing on the left hand. The finger tapping test score is generated by an average of the scores on the left and the right hand.

### 4.8. Symbol Digit Coding (SDC)

The symbol digit coding test will first give the participant a training session on how the numbers are linked to digits (Gualtieri & Johnson, 2006) [62]. The test then will begin, showing one screen at a time with 8 symbols and 8 empty boxes below. The aim is for the participant to type the corresponding digit (from 2 to 9) in the empty box. The test will continue showing 8 symbols and 8 empty boxes per screen for 120 s. The score for the symbol digit coding test is generated by using the correct number of responses in 2 min.

The number of right and left taps from the finger tapping test, and the number of correct responses from the symbol digit coding test are combined to form a score for psychomotor speed.

### 4.9. The Stroop Test (ST)

The Stroop Test that Vital Signs use consists of four colors/colored words and the space bar (Gualtieri & Johnson, 2006) [62]. The test is made up of three sections. In the first section, the words red, yellow, blue and green appear in black text at random, and the participant should press the space bar as soon as they appear. This will generate the score for simple reaction time. In the next section, the same words appear but this time in different colors. The participant is asked to tap the space bar when the color of the word matches the word. This part of the test generates a complex reaction time score. The final part of the test is the same as the second, but instead the participant is asked to tap the space bar when the color of the word does not match the word. This also generates a complex reaction time score, called the color-word reaction time. An error score is also created. Taking an average of the scores from Section 2 and Section 3 gives a score for processing speed.

### 4.10. The Shifting Attention Test (SAT)

The shifting attention test is designed to measure a participant’s ability to shift from one set of instructions to another accurately (Gualtieri & Johnson, 2006) [62]. In the test, the participants are asked to match geometric objects by either shape or color when three figures appear on a screen. The images are placed with one on the top (either a square or circle), and two on the bottom (a square and circle each). All figures are either red or blue. The top figure is the one that will need to be matched to either figure on the bottom. The rules for matching this are random and do change. This test goes on for 90 s with the goal of choosing the correct match as many times as the participant can. The score for cognitive flexibility can be generated by subtracting the number of response errors on the SAT and Stroop Test, from the number of correct responses.

### 4.11. The Continuous Performance Test (CPT)

The Continuous Performance Test in Vital Signs asks the participant to respond to the letter “B” which is the target stimulus, but not any other letter (Gualtieri & Johnson, 2006) [62]. In a timeframe of 5 min, the participant will see 200 letters, of which 40 are the target letter. The letters are shown randomly, with the target stimulus being shown 8 times per minute. The test is scored into three categories: correct responses, commission errors, and omission errors. The test will also report the participant’s reaction time to choose for each variable. The score for complex attention is generated by adding the number of errors committed in the CPT, the SAT, and the Stroop.

### 4.12. Statistical Analyses

Data were processed in Microsoft Excel 2023 and GraphPad Prism software version 10 was used for all statistical analyses, including the calculation of serum and exosome concentrations, means, standard deviations (SDs), coefficient of determination (R^2^), and *p* values (where * significant *p* < 0.05; ** highly significant *p* < 0.01; n.s. denotes non-significant). The test carried out was a Mann–Whitney U test. All quantitative data are expressed as mean values ± SD of the mean for individual cases.

## 5. Conclusions

This study highlights the potential of lipid biomarkers, particularly 24-HC, 27-HC, ceramide, and triglycerides, as indicators of concussion-related neurodegenerative risk in retired rugby players. The observed alterations in lipid levels mirror patterns reported in neurodegenerative diseases such as AD, PD, and ALS, reinforcing the biological relevance of these markers. While 25-HC showed no significant difference, its elevation in some concussed individuals warrants further exploration. These findings support the use of lipid profiling as a non-invasive approach to detect early neurodegenerative changes, offering valuable insight for clinical monitoring, early intervention, and refining concussion management protocols in high-impact sports.

## Figures and Tables

**Table 1:** Descriptive characteristics.

	Concussed Group N = 26	Control Group N = 19	*p* Values
**Mean ages**	39.32 ± 6.44	47.19 ± 12.11	0.0308
**Mean ages at retirement**	26.56 ± 4.89	33.77 ± 9.24	0.0622
**Mean years since retirement from the sport**	7.04 ± 5.23	8.33 ± 4.29	0.4957
**Playing position**	4 prop, 3 hooker, 1 forward, 2 s row, 1 fly half, 4 center, 4 wing, 3 backward, 1 openside flanker, 1 blindside flanker, 2 lock.	1 blindside flanker, 2 backwards, 1 wing, 1 number 8, 1 prop, 1 standoff, 12 non-athletes.	
**Mean weight (kg)**	100.2 ± 11.14	86.53 ± 15.47	0.0083
**Mean height (cm)**	183.81 ± 7.09	178.5 ± 6.33	0.0257
**BMI**	29.56 ± 3.64	27.4 ± 4.10	0.0829
**Rugby league (RL) or union (RU)**	12 (RL), 14 (RU)	5 (RU), 2 (RL), 12 (N/A)	

**Table 2:** Comparison of cognitive function between the two groups. Comparison between a group who has sustained 5 or more concussions and a group that has suffered from none in career. * = *p* < 0.05.

		≥5 Concussions	No Concussions	*p* Value
NCI		89.83 ± 13.44	104 ± 2.16	0.049 (*)
Memory		91.70 ± 16.81	110.25 ± 8.73	0.076
Psychomotor speed		97.43 ± 14.46	106.25 ± 7.37	0.249
Reaction time		86.04 ± 27	91.50 ± 14.55	0.700
Complex attention		86.57 ± 24.10	109 ± 3.16	0.079
Cognitive flexibility		87.35 ± 15.74	104 ± 5.03	0.049 (*)
Processing speed		97.32 ± 15.46	110.25 ± 9.22	0.061
Executive function		87.86 ± 15.24	104 ± 5.48	0.025 (*)
Simple attention		88.70 ± 24.44	99 ± 14.67	0.215

## Data Availability

The original contributions presented in this study are included in the article. Further inquiries can be directed to the corresponding authors.
